# CYP1B1 affects the integrity of the blood–brain barrier and oxidative stress in the striatum: An investigation of manganese‐induced neurotoxicity

**DOI:** 10.1111/cns.14633

**Published:** 2024-03-01

**Authors:** Juan Wu, Yueran Li, Shuwei Tian, Shufang Na, Hongyan Wei, Yafei Wu, Yafei Yang, Zixia Shen, Jiayue Ding, Shenglan Bao, Siqi Liu, Lingyun Li, Rongling Feng, Yong Zhu, Chunyan He, Jiang Yue

**Affiliations:** ^1^ Department of Pharmacology, School of Basic Medical Sciences Wuhan University Wuhan China; ^2^ Department of Pharmacy Taikang Tongji (Wuhan) Hospital Wuhu China; ^3^ Department of Pharmacy The First Affiliated Hospital of Wannan Medical College Wuhu China; ^4^ Zhongnan Hospital of Wuhan University, Institute of Hepatobiliary Diseases of Wuhan University Transplant Center of Wuhan University, Hubei Key Laboratory of Medical Technology on Transplantation Wuhan Hubei China; ^5^ Demonstration Center for Experimental Basic Medicine Education, School of Basic Medical Sciences Wuhan University Wuhan China; ^6^ Hubei Province Key Laboratory of Allergy and Immunology Wuhan China

**Keywords:** blood–brain barrier, CYP1B1, dopamine, manganese, MAO‐B, neurotoxicity

## Abstract

**Aims:**

Excessive influx of manganese (Mn) into the brain across the blood–brain barrier induces neurodegeneration. CYP1B1 is involved in the metabolism of arachidonic acid (AA) that affects vascular homeostasis. We aimed to investigate the effect of brain CYP1B1 on Mn‐induced neurotoxicity.

**Method:**

Brain Mn concentrations and α‐synuclein accumulation were measured in wild‐type and CYP1B1 knockout mice treated with MnCl_2_ (30 mg/kg) and biotin (0.2 g/kg) for 21 continuous days. Tight junctions and oxidative stress were analyzed in hCMEC/D3 and SH‐SY5Y cells after the treatment with MnCl_2_ (200 μM) and CYP1B1‐derived AA metabolites (HETEs and EETs).

**Results:**

Mn exposure inhibited brain CYP1B1, and CYP1B1 deficiency increased brain Mn concentrations and accelerated α‐synuclein deposition in the striatum. CYP1B1 deficiency disrupted the integrity of the blood–brain barrier (BBB) and increased the ratio of 3, 4‐dihydroxyphenylacetic acid (DOPAC) to dopamine in the striatum. HETEs attenuated Mn‐induced inhibition of tight junctions by activating PPARγ in endothelial cells. Additionally, EETs attenuated Mn‐induced up‐regulation of the KLF/MAO‐B axis and down‐regulation of NRF2 in neuronal cells. Biotin up‐regulated brain CYP1B1 and reduced Mn‐induced neurotoxicity in mice.

**Conclusions:**

Brain CYP1B1 plays a critical role in both cerebrovascular and dopamine homeostasis, which might serve as a novel therapeutic target for the prevention of Mn‐induced neurotoxicity.

## Graphical abstract

CYP1B1 deficiency disrupts the integrity of the blood–brain barrier and aggravates manganese‐induced neurotoxicity in the striatum. Biotin may play a protective role against manganese‐induced neurotoxicity by the up‐regulation of CYP1B1.

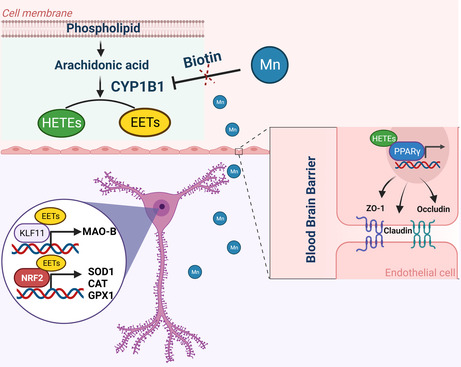

## 1 INTRODUCTION

Inhalation of manganese (Mn) fumes or dust has been recognized as a huge concern in occupational and environmental health. Mn is an indispensable trace metal for brain development, but excessive Mn deposition in the brain results in neurological disorders (e.g., Parkinsonism).^1^ The divalent Mn has been shown to induce the liquid‐to‐solid phase transition of α‐synuclein and facilitate the aggregation of amyloid.^2^ The high accumulation of Mn was observed in the striatum, globus pallidus, ventral pallidum, and substantia nigra in mice intravenously injected with MnCl_2_·4H_2_O.^3^ The clearance of Mn from the brain is very slow, and the half‐lives range from 51 to 74 days in various regions.^4^ The temporary disruption of the blood–brain barrier (BBB) by ultrasound or hyperosmotic mannitol effectively increased the entrance of Mn into rodent brains.^5^, ^6^, ^7^ Previous data indicated that one or more transporters could be involved in the uptake of Mn in the brain, while divalent Mn may also diffuse into the brain as an ionic or as a nonspecific binding type.^8^

The cytochrome P450 (CYP) superfamily primarily located in the endoplasmic reticulum and mitochondrial membrane plays an important role in the synthesis and metabolism of endogenous and exogenous substances. The metabolites of arachidonic acid (AA) catalyzed by cytochrome P450 (CYP) play a key role in cardiovascular biology, especially hydroxyeicosatetraenoic acids (HETEs) and epoxyeicosatrienoic acids (EETs).^9^ CYP1B1, an extrahepatic isoform, has been shown to be involved in AA metabolism in both the cortex and the cerebellum.^10^ CYP1B1 is abundant in brain microvascular, accounting for 77% of all CYP isoforms expressed in brain microvascular.^11^, ^12^ β‐catenin signaling is crucial for the development and maintenance of cerebral blood vessels,^13^ while CYP1B1 was significantly decreased in endothelial cells with β‐catenin deficiency.^14^ However, the involvement of brain CYP1B1 in toxin‐induced neurotoxicity remains unclear. We investigated the involvement of CYP1B1 in Mn‐induced neurotoxicity in mice. The present study adds to the current knowledge on the influence of brain CYP1B1 in toxin‐induced neurotoxicity.

## 2 METHODS

### 2.1 Animals and treatment

Male adult CYP1B1 knockout mice kindly provided by Dr. Frank J. Gonzalez,^15^ and their wild‐type counterparts (C57BL/6J) were bred at the Centre for Animal Experiments Laboratory of Wuhan University. The mice were genotyped using DNA samples extracted from the tails. All the animals were kept in a room (22 ± 2°C) under a 12 h artificial light/dark cycle with free access to food and water. All procedures were approved by the Animal Care Committee of Wuhan University.

Male adult mice were intranasally instilled with MnCl_2_ (2 μL, 30 mg/kg) or distilled water for 21 continuous days as previously described.^16^, ^17^ To investigate the effects of biotin on Mn‐induced neurotoxicity, mice were intragastrically administrated with biotin (0.2 g/kg) or vehicle following Mn treatment for 30 min.

### 2.2 Flame atomic absorption spectrophotometry

Animals were sacrificed by decapitation 2 h after the last treatment and the brains were perfused with saline to remove blood. The sample was mixed with 2 mL of concentrated nitric acid and 0.5 mL of 30% hydrogen peroxide and then heated in an oil bath at 140°C. After the evaporation of acid, the samples were diluted to 10 mL with ultrapure water. A high‐resolution continuum source atomic absorption spectrometer (contrAA700) was used as an instrumental detection system with a Xenon short‐arc lamp and air/acetylene burner head.^18^, ^19^ The wavelength used for Mn was 279.5 nm.

### 2.3 Behavioral experiment

The open‐field test is used to assess the general activity of mice. Each mouse was placed in the center of the arena (42 × 42 × 30 cm, AccuScan Instruments), and was allowed to move freely. The total distance traveled within 5 min was recorded using the SMART video tracking system.^20^

The rotarod test is used to evaluate the motor balance and coordination of mice. The mice were trained for three consecutive days, and the rotating speed was increased from 4 to 40 rpm for 3 min training sessions. In the final experiment, the mice were placed on the rod, and their falling time was recorded within 10 min. The experiment was repeated three times for each mouse with a minimum interval of 30 min, and the mean duration was used as the result.

### 2.4 In vivo multiphoton microscopy assay

Male mice (5‐ and 12‐month old) were anesthetized by the intraperitoneal injection (50 mg/kg) with sodium pentobarbital and then were secured in a stereotaxic frame (RWD Life science, Shenzhen, China). A 6 mm in diameter cranial window over the parietal cortex was made by a cranial drill,^21^ and the skull was removed while keeping the dural integrity. Imaging was completed within 40 min as previously described, and no significant inflammation or gliosis was observed.^22^ FITC‐dextran (70 kDa, sigma, 0.2 mL of 10 mg/kg) was injected via the tail vein, and the images were captured by an A1SiMP multiphoton microscope. Multiphoton z‐stack images were taken from the depth of 50 μm below the cortical brain surface to 500 μm with an interval of 2 μm between each acquisition. The cortical microvascular length (diameter <6 μm) in diameter was determined within the 240 × 240 × 10 μm field by a blinded investigator using the Image J software (National Institutes of Health, Bethesda, MD, USA). The average capillary length was calculated from 12 images per mouse, and three mice per group were analyzed.

### 2.5 Immunofluorescence

Brain sections were incubated with a primary antibody overnight at 4°C after blocking. The primary antibodies were as follows: a monoclonal mouse anti‐mouse α‐synuclein antibody (1:400); a polyclonal rabbit anti‐mouse tyrosine hydroxylase antibody (1:500); a polyclonal rabbit anti‐mouse ZO‐1 antibody (1:400), a monoclonal rabbit anti‐mouse Fibrin antibody (1:200), and a monoclonal mouse anti‐human CD31 antibody (1:200). As secondary antibodies, a rhodamine‐conjugated AffiniPure Goat anti‐rabbit IgG for tyrosine hydroxylase (1:100) and a Cy3‐conjugated AffiniPure Donkey anti‐mouse IgG for α‐synuclein (1:100) were used. Images were analyzed with an Olympus BX 51 fluorescence microscope (Olympus Corporation, Tokyo, Japan) equipped with an Olympus Micro DP 72 camera. The identical settings were used for each image dataset. We used Image J (National Institutes of Health, Bethesda, MD, USA) to calculate the integrated option density (IOD).

### 2.6 Real‐time RT‐PCR

Total RNA from the cells or brain regions was extracted using TRIzol Reagent (Invitrogen, CA, USA) in accordance with the manufacturer's protocol. cDNA was synthesized with a cDNA Synthesis Kit (Toyobo, Osaka, Japan) for first‐strand synthesis. The real‐time RT‐PCR reactions with SYBR Green (Toyobo, Osaka, Japan) were performed using a CFX connect real‐time PCR detection system (Bio‐Rad, Hercules, CA, USA). The primers and PCR conditions are listed in Table S1. The relative expression levels were normalized by GAPDH and were calculated using the 2^−△△^Ct method.

### 2.7 UPLC‐MS/MS analysis

All of the eicosanoid standards including HETEs, EETs, and dihydroxy‐eicosatrienoic acids (DHETs) were purchased from Cayman Chemical (Arbor, MI, USA). AA metabolites in the striatum were detected as previously described.^23^ Briefly, samples were homogenized and extracted with an extraction solvent containing 2,6‐di‐tert‐butyl‐4‐methylphenol and formic acid. The stable isotope probes, 2‐dimethylaminoethylamine (DMED) and d4‐DMED were added for derivatization. A Shimadzu LC‐30 AD UPLC (Tokyo, Japan) equipped with an Acquity UPLC BEH phenyl column (2.1 mm × 50 mm, 1.7 m, Waters) was used for UPLS analysis. The separation was performed with the mobile phase consisting of (A) FA in water (0.1%, v/v) and (B) ACN/MeOH (7/3, v/v). An ABI/SCIEX 4500 Triple Quad™ equipped with a Turbo V ion source was used for mass spectrometry analysis. Samples were detected using multiple reaction monitoring mode.

### 2.8 Cell culture and treatment

The human cerebral microvascular endothelial cell line (hCMEC/D3) was cultured in DMEM/F12 medium. Human neuroblastoma SH‐SY5Y cells were maintained in the DMEM medium. The cells were treated with MnCl_2_ (200 μM), 5‐HETE (1 μM), 15‐HETE (1 μM), 12‐(3‐adamantan‐1‐yl‐ureido)‐dodecanoic acid (AUDA) (1 μM), and 14,15‐EET (1 μM). For the co‐treatment, MnCl_2_ and AA metabolites were added into the medium without fetal bovine serum at the same time, and AUDA was administrated 1 h before 14,15‐EET and MnCl_2_. To investigate the effects of biotin on KLF11 and MAO‐B, SH‐SY5Y cells were treated with biotin (40 μM) or vehicle for 24 h.

### 2.9 Transient transfection and luciferase assay

The fragments encoding CYP1B1 and KLF11 were cloned into the pcDNA3.1 (+) vector. The fragment encoding PPARγ was cloned into the pSG5 vector. The luciferase reporter PPAR response element (PPRE) X3‐TK‐Luc was obtained from Addgene (Watertown, MA). All the constructs were verified by DNA‐sequence analysis. To investigate the regulation of tight junctions by CYP1B1, hCMEC/D3 cells were transiently transfected with CYP1B1 or PPARγ expression vector for 24 h. To investigate the regulation of MAO‐B by CYP1B1, SH‐SY5Y cells were transiently transfected with the CYP1B1 or KLF11 expression vector for 24 h. The respective empty vectors were used as the control.

To investigate the effects of HETEs on PPARγ transcriptional activity, hCMEC/D3 cells were treated with 5‐HETE (1 μM) and 15‐HETE (1 μM) following co‐transfection with PPRE luciferase reporter and PPARγ expression vector for 24 h. The luciferase activity was determined using the Dual‐Luciferase Reporter Assay kit (Promega, Madison, WI, USA), and the firefly luciferase activity was normalized to the Renilla luciferase activity.

### 2.10 Statistical analysis

Relative mRNA and protein levels were expressed as arbitrary units. Age‐ and sex‐matched wild‐type and CYP1B1 knockout mice were assigned to groups without randomization. The cellular data were obtained from at least three independent cell preparations. The data from in vivo experiments are shown as the mean ± SD, and the data from in vitro experiments are shown as the mean ± SEM. The Shapiro–Wilk test was used to test normality. The differences between the two treatment groups were tested with an unpaired two‐tailed Student's *t‐*test. The differences among the groups were analyzed by one‐way ANOVA followed by the least significant difference (LSD) test for equal variances or Tamhane's T2 for unequal variances. Differences with *p* < 0.05 were considered significant.

## 3 RESULTS

### 3.1 CYP1B1 deficiency accelerates Mn accumulation in brain tissue and aggravates Mn‐induced damage to dopaminergic neurons

**FIGURE 1:**
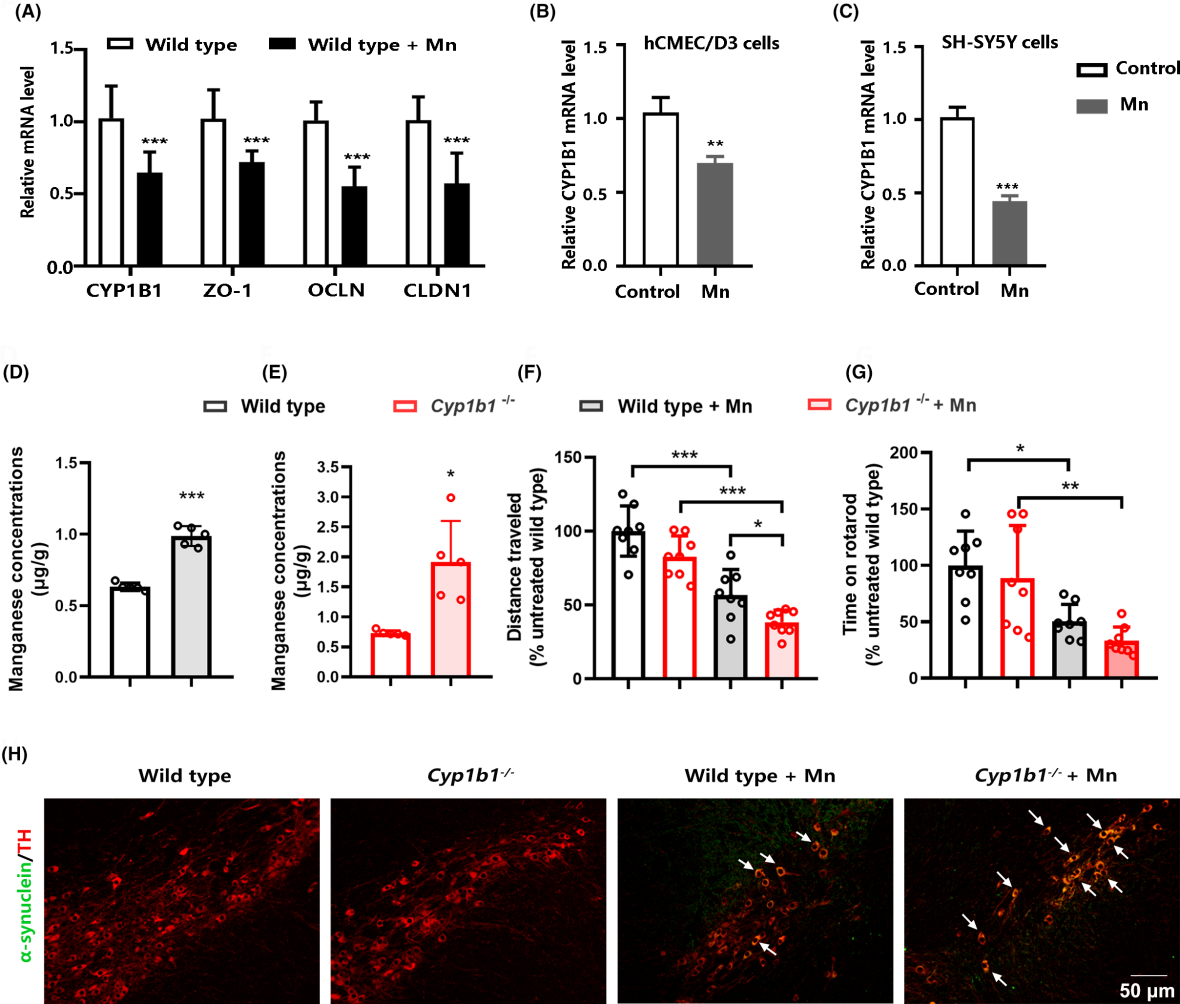
Mn‐induced neurotoxicity is exacerbated by CYP1B1 deficiency. (A) Relative mRNA levels of CYP1B1 and genes related to tight junctions (ZO‐1, OCLN, and CLDN1) in the striatum treated with Mn (*n* = 5). (B) Relative mRNA levels of CYP1B1 in hCMEC/D3 cells treated with Mn (*n* = 3). (C) Relative mRNA levels of CYP1B1 in SH‐SY5Y cells treated with Mn (*n* = 3). (D) Brain Mn concentration in 2‐month‐old wild‐type mice intranasally exposed to Mn for 21 days (*n* = 5). (E) Brain Mn concentration in 2‐month‐old CYP1B1 knockout mice intranasally exposed to Mn for 21 days (*n* = 5). (F) The total distance traveled within 5 min in the open‐field test (*n* = 8). (G) Time spent on the rotarod in rotarod test (*n* = 8). (H) Representative images of α‐synuclein (green) and tyrosine hydroxylase‐positive (TH) neurons (red) in the substantia nigra (*n* = 3). Data in (B) and (C) are shown as the mean ± SEM; data in all other panels are shown as the mean ± SD. An unpaired two‐tailed Student's *t‐*test was used to compare the means between two groups, and one‐way ANOVA followed by the least significant difference (LSD) was used to identify the differences among four groups; *p < 0.05, **p < 0.01, ***p < 0.001.

The mRNA levels of CYP1B1 and tight junction proteins (ZO‐1, OCLN, and CLDN1) were reduced in the striatum from the wild‐type mice exposed to Mn, compared with the control (Figure 1A). Given that CYP1B1 is highly expressed in brain microvessels and neuronal cells, we observed the effects of Mn exposure on CYP1B1 levels in endothelial and neuronal cells, respectively. Mn exposure reduced mRNA levels of CYP1B1 in both endothelial hCMEC/D3 cells and neuroblastoma SH‐SY5Y cells (Figure 1B,C). We found that wild‐type mice had increased Mn concentrations in whole brain tissue after intranasal Mn exposure (Figure 1D). Compared with wild‐type mice, CYP1B1 knockout mice showed an accelerated accumulation of Mn in brain tissue after Mn exposure (Figure 1E), which may be owing to the increased BBB permeability by CYP1B1 deficiency. The data from the open‐field test and rotarod test revealed that Mn exposure reduced the locomotor activity and motor balance capability in wild‐type mice (Figure 1F,G). CYP1B1 knockout mice had a shortened total distance and duration on rotarod after Mn exposure compared with the control, indicating that CYP1B1 deficiency exacerbated motor disorders. The accumulation of α‐synuclein in dopaminergic neurons was observed in the substantia nigra from wild‐type mice after Mn exposure, and CYP1B1 deficiency aggravated the accumulation of α‐synuclein (Figure 1H).

### 3.2 CYP1B1 deficiency drives the reduction of brain microcirculation and disruption of tight junctions

**FIGURE 2:**
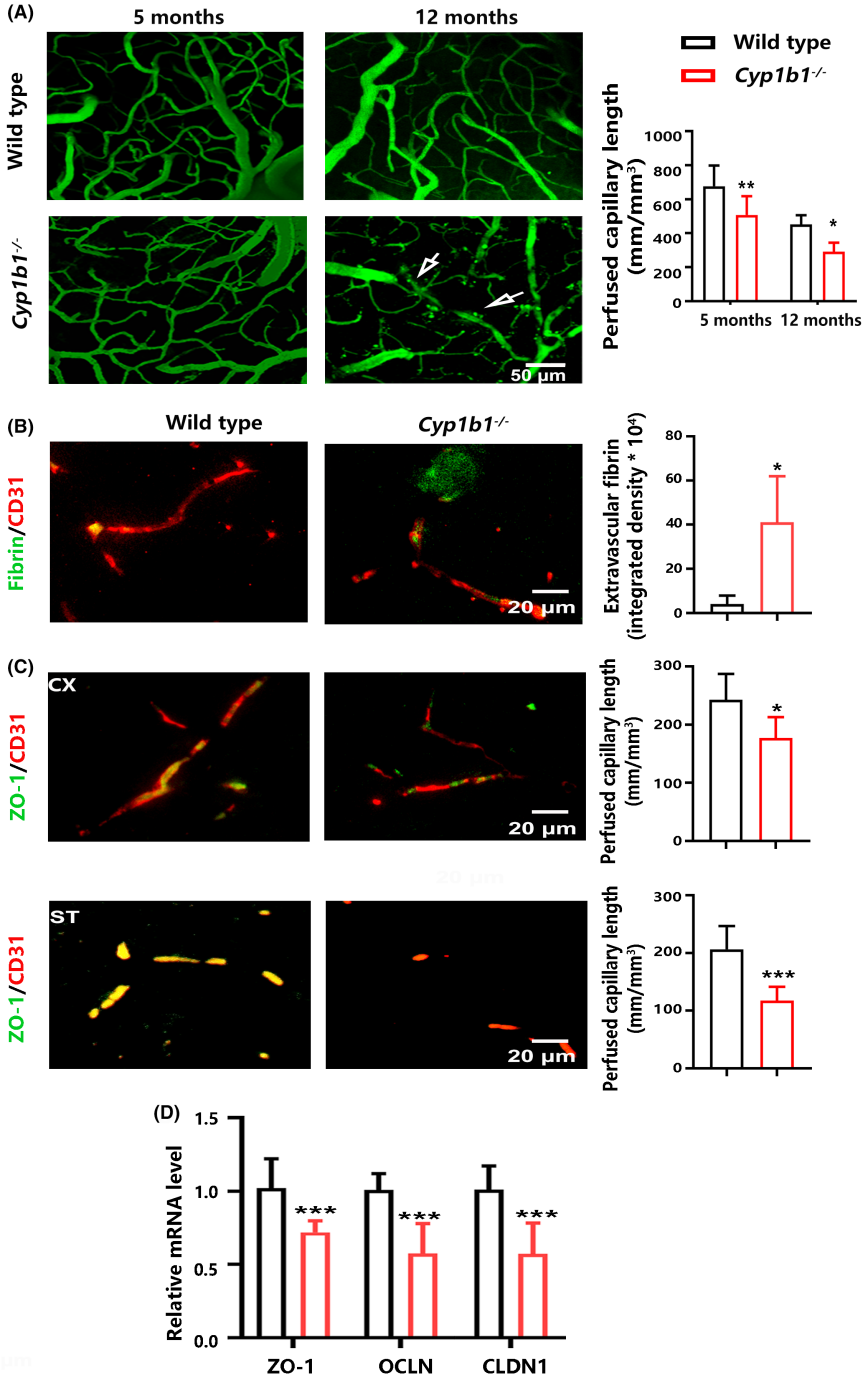
Changes in brain microcirculation and tight junctions of BBB in CYP1B1 knockout mice. (A) Representative in vivo multiphoton microscopy analysis of FITC‐dextran (70 kDa) in the parietal cortex from 5‐ and 12‐ month‐old mice (*n* = 4). (B) Representative images of fibrin (green) and CD31‐positive microvessels (red) in the parietal cortex from 12‐month‐old mice (*n* = 4). (C) Co‐localization analysis of ZO‐1 proteins (green) and CD31‐positive microvessels (red) in the cortex (CX) and striatum (ST) from 12‐month‐old mice (*n* = 4). (D) Relative mRNA levels of ZO‐1, CLDN1, and OCLN in the ST from 2‐month‐old mice (*n* = 5). All data are shown as the mean ± SD. An unpaired two‐tailed Student's *t‐*test; *p < 0.05, ***p < 0.001.

The in vivo images have shown that the length of microvessels in the parietal cortex was reduced in CYP1B1 knockout mice at the age of 5 and 12 months, compared with wild‐type mice (Figure 2A). The extravasation of 70 kDa FITC‐dextran in the parietal cortex was observed in 12‐month‐old *Cyp1b1* ^*−/−*^ mice (Figure 2A), indicating the disruption of BBB. The increase in co‐localization of vascular marker CD31 and fibrin confirmed BBB disruption in the cortex from 12‐month‐old *Cyp1b1* ^−/−^ mice (Figure 2B). Compared with wild‐type mice, the reduced levels of tight junction protein ZO‐1 were observed in microvessels of both the cortex and the striatum from 12‐month‐old *Cyp1b1* ^−/−^ mice (Figure 2C); additionally, the mRNA levels of tight junction proteins (ZO‐1, OCLN, and CLDN1) were decreased in the striatum from 2‐month‐old *Cyp1b1* ^−/−^ mice (Figure 2D). The data suggest that brain CYP1B1 may affect BBB structure and permeability.

### 3.3 CYP1B1‐derived HETEs attenuate Mn‐induced inhibition of tight junctions via the activation of PPARγ in endothelial cells

**FIGURE 3:**
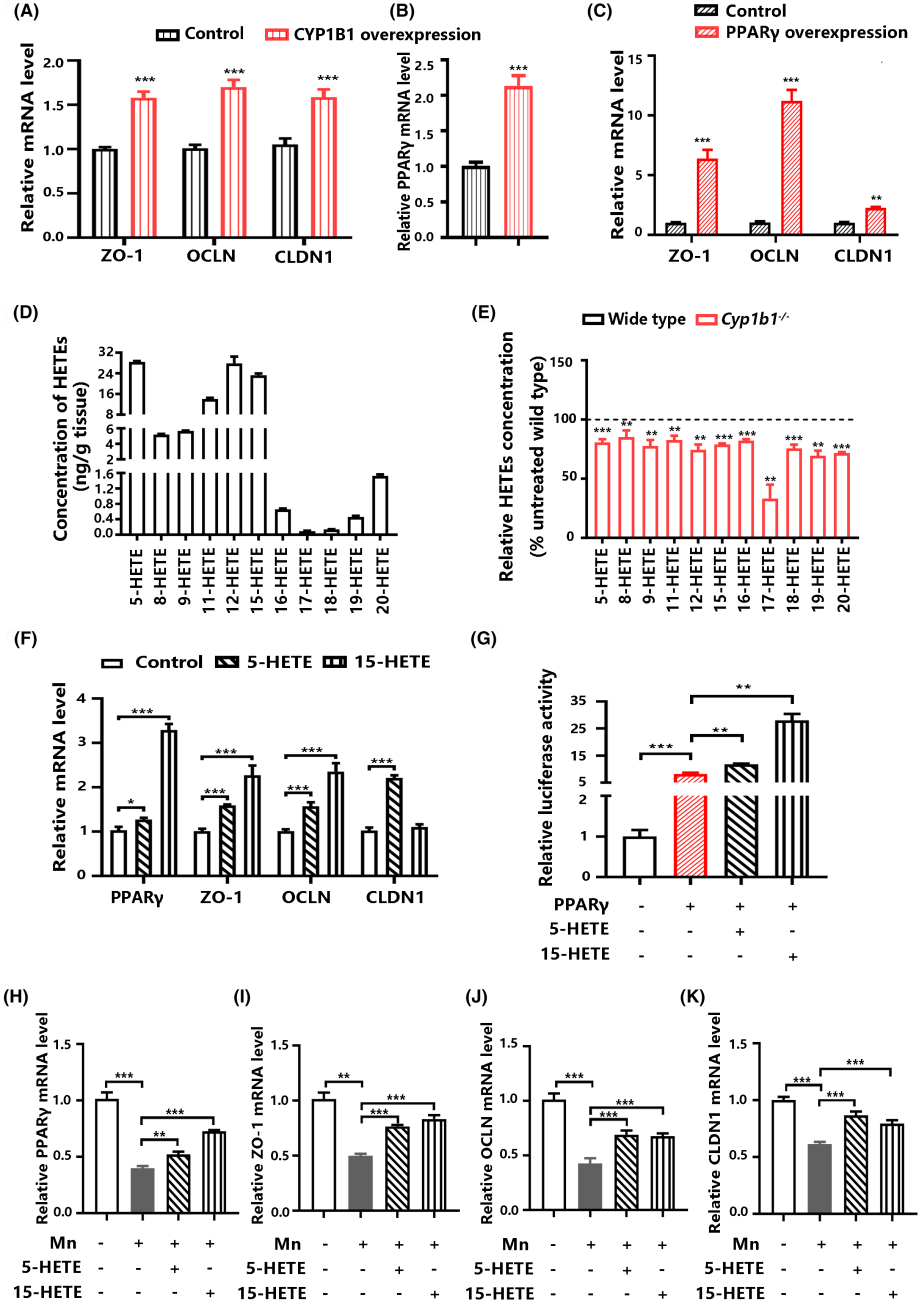
CYP1B1‐derived HETEs attenuate Mn‐induced inhibition of tight junctions in endothelial cells. (A) Relative mRNA levels of genes related to tight junctions (ZO‐1, OCLN, and CLDN1) in hCMEC/D3 cells transfected with the CYP1B1 expression vector (*n* = 3). (B) Relative PPARγ mRNA level in hCMEC/D3 cells transfected with the CYP1B1 expression vector (*n* = 3). (C) Relative mRNA levels of genes related to tight junctions in hCMEC/D3 cells transfected with PPARγ expression vector (*n* = 3). (D) The concentrations of HETEs in the striatum from the wild‐type mice (*n* = 3). (E) Changes in the concentrations of HETEs in the striatum from *Cyp1b1* ^−/−^ mice relative to the wild‐type mice (*n* = 3). (F) Relative mRNA levels of PPARγ and genes related to tight junctions in hCMEC/D3 cells exposed to HETEs (*n* = 3). (G) The luciferase activity in hCMEC/D3 cells transfected with the PPRE luciferase reporter vector. Basal activity levels measured in cells transfected with the empty vector were set to 1 (*n* = 3). (H‐K) Relative mRNA level of PPARγ and genes related to tight junctions in hCMEC/D3 cells exposed to Mn and HETEs (*n* = 3). Data in (D) and (E) are shown as the mean ± SD; data in all other panels are shown as the mean ± SEM. Data in (A), (B), (C), and (E) were analyzed using an unpaired two‐tailed Student's *t*‐test; data in (F), (J), and (K) were analyzed using one‐way ANOVA followed by the least significant difference (LSD); data in (G), (H), and (I) were analyzed using one‐way ANOVA followed by Tamhane's T2; **p < 0.01, ***p < 0.001.

Unsurprisingly, mRNA levels of tight junction proteins (ZO‐1, OCLN, and CLDN1) were increased in hCMEC/D3 cells by CYP1B1 overexpression, compared with the control (Figure 3A). A previous study has shown that PPARγ agonists strengthen tight junctions of human nasal epithelial cells.^24^ CYP1B1 overexpression increased PPARγ mRNA levels in hCMEC/D3 cells, compared with the control (Figure 3B). Additionally, PPARγ overexpression up‐regulated the mRNA levels of tight junction proteins (Figure 3C). The data suggest the involvement of PPARγ in the regulation of BBB permeability by CYP1B1.

We observed the effect of endogenous metabolites on tight junctions in endothelial cells. The major HETEs generated from AA in the striatum from the wild‐type mice included 5‐HETE, 11‐HETE, 12‐HETE, and 15‐HETE (Figure 3D). CYP1B1 deficiency resulted in decreases in most HETEs in the striatum (Figure 3E). The mRNA levels of PPARγ and tight junctions were increased in hCMEC/D3 cells treated with CYP1B1‐derived AA metabolites, 5‐HETE and 15‐HETE, compared with the control (Figure 3F); meanwhile, 5‐HETE and 15‐HETE enhanced the transcriptional activity of PPARγ in these cells (Figure 3G). The data suggest that CYP1B1‐derived HETEs may be involved in the regulation of tight junctions in the brain.

We also observed the effect of CYP1B1‐derived metabolites on Mn‐induced inhibition of tight junctions. The Mn‐induced reduction of PPARγ and genes related to tight junctions was attenuated by 5‐HETE and 15‐HETE (Figure 3H–K). The data suggest that up‐regulation of brain CYP1B1 may attenuate the disruption of tight junctions by Mn exposure.

### 3.4 CYP1B1 deficiency aggravates Mn‐induced oxidative stress

**FIGURE 4:**
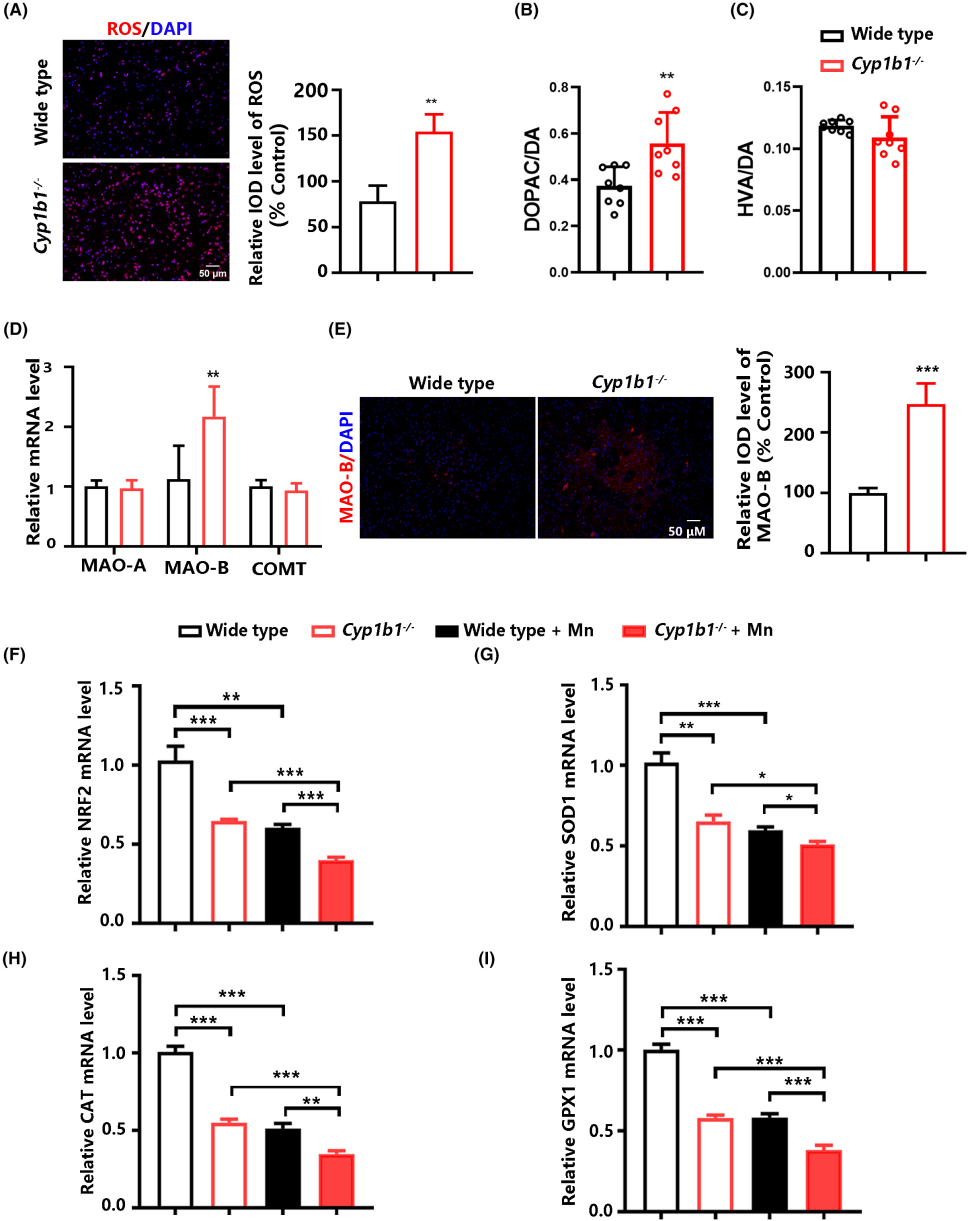
CYP1B1 deficiency exacerbates oxidative stress after Mn exposure. (A) Changes in ROS levels in the striatum from *Cyp1b1* ^*−/−*^ mice relative to the wild‐type mice (*n* = 4). (B) The ratio of 3,4‐dihydroxyphenylacetic acid (DOPAC) to dopamine in the striatum (*n* = 8). (C) The ratio of homovanillic acid (HVA) to dopamine in the striatum (*n* = 8). (D) Relative mRNA levels of MAO‐A, MAO‐B, and COMT in the striatum (*n* = 4). (E) Representative images of MAO‐B (red) in the striatum (*n* = 3). (F‐I) Relative mRNA levels of genes related to mitochondrial oxidative defense including NRF2, SOD1, CAT, and GPX1 in the striatum (*n* = 5). All data are shown as the mean ± SD. Data in (A), (B), (C), (D), and (E) were analyzed using an unpaired two‐tailed Student's *t*‐test; data in (F), (H), and (I) were analyzed using one‐way ANOVA followed by the least significant difference (LSD); data in (G) were analyzed using one‐way ANOVA followed by Tamhane's T2; *p < 0.05, **p < 0.01, ***p < 0.001.

Previous data have shown that oxidative stress played a critical role in Mn‐induced neurotoxicity.^25^ We found that reactive oxygen species (ROS) levels were increased in the striatum from *Cyp1b1* ^−/−^ mice, compared with the control (Figure 4A).

Monoamine oxidase B (MAO‐B) and catechol‐O‐methyltransferase (COMT) are the main enzymes responsible for dopamine metabolism in the brain. The degradation of dopamine by MAO‐B is accompanied by ROS production. A previous study has shown that the transgenic mice with the specific elevation of astrocytic MAO‐B levels were more sensitive to toxin‐induced damage to dopaminergic neurons due to the increased mitochondrial oxidative stress.^26^ We found that the ratio of 3,4‐dihydroxyphenylacetic acid (DOPAC) to dopamine was increased in the striatum from *Cyp1b1* ^−/−^ mice, compared with the control (Figure 4B). However, the ratio of homovanillic acid (HVA) to dopamine was unchanged by CYP1B1 deficiency (Figure 4C). The mRNA and protein levels of MAO‐B were up‐regulated in the striatum from *Cyp1b1* ^−/−^ mice compared with the control, while no changes in MAO‐A, and COMT were observed (Figure 4D,E).

A previous study has shown that Mn preferentially enters into mitochondria.^27^ Nuclear factor erythroid 2‐related factor 2 (NRF2) is essential for mitochondrial ROS homeostasis by binding to the antioxidant response element (ARE) of oxidative defense genes.^28^ The mRNA levels of NRF2 and its target genes (SOD1, CAT, and GPX1) in the striatum from Mn‐treated wild‐type mice were decreased, and CYP1B1 deficiency aggravated the reduction of these genes (Figure 4F–I).

### 3.5 CYP1B1‐derived EETs attenuate Mn‐induced up‐regulation of the KLF/MAO‐B axis and down‐regulation of NRF2 in neuronal cells

**FIGURE 5:**
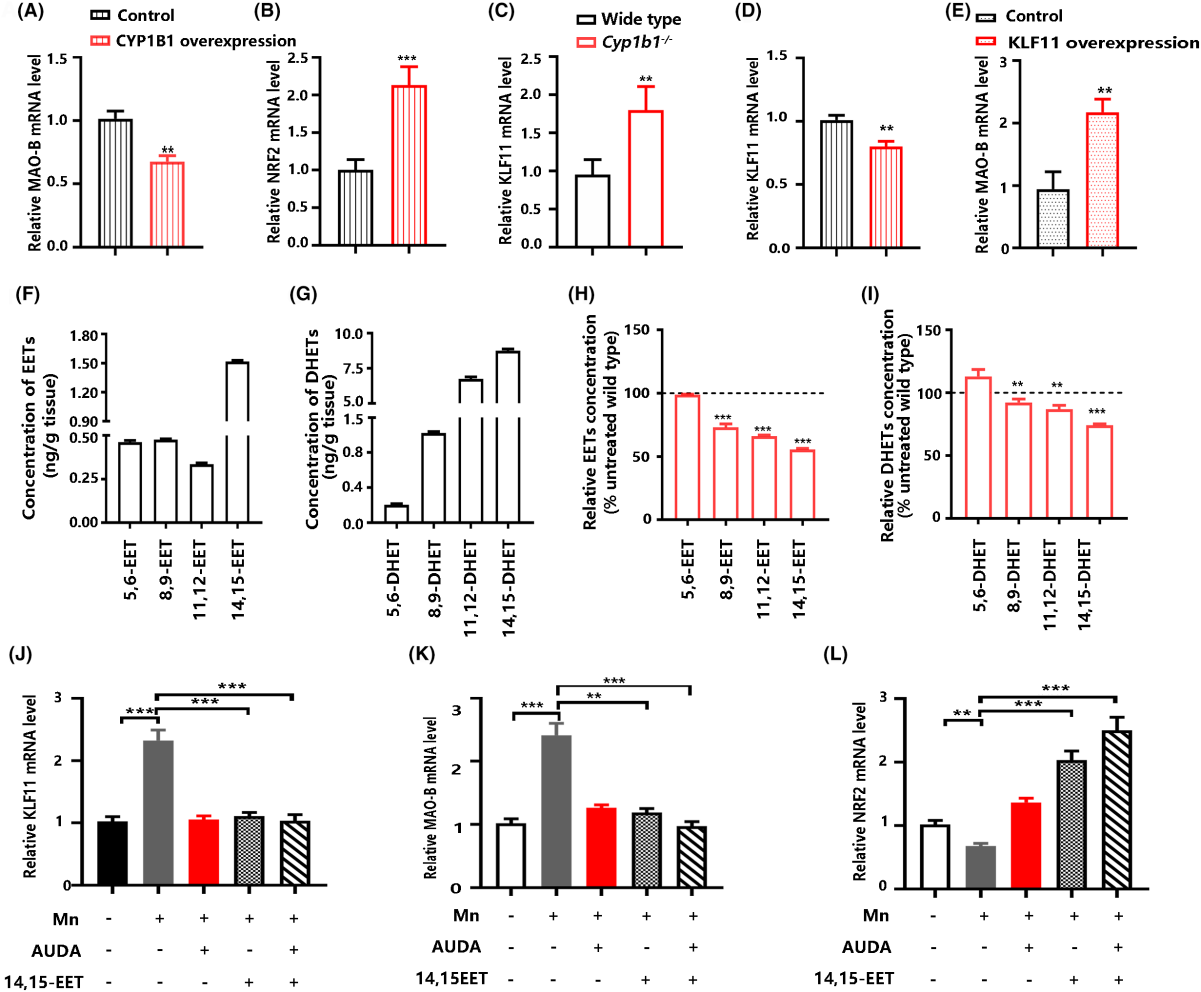
CYP1B1‐derived EETs attenuate Mn‐induced oxidative stress in neuronal cells. (A) Relative MAO‐B mRNA level in SH‐SY5Y cells transfected with the CYP1B1 expression vector (*n* = 3). (B) Relative NRF2 mRNA level in SH‐SY5Y cells transfected with the CYP1B1 expression vector (*n* = 3). (C) Relative KLF11 mRNA level in the striatum (*n* = 4). (D) Relative KLF11 mRNA level in SH‐SY5Y cells transfected with the CYP1B1 expression vector (*n* = 3). (E) Relative MAO‐B mRNA level in SH‐SY5Y cells transfected with the KLF11 expression vector (*n* = 3). (F‐G) The concentrations of EETs and DHETs in the striatum from the wild‐type mice (*n* = 3). (H‐I) Changes in the concentrations of EETs and DHETs in the striatum from *Cyp1b1* ^−/−^ mice relative to the wild‐type mice (*n* = 3). (J‐M) Relative mRNA levels of KLF11, MAO‐B, and NRF2 in SH‐SY5Y cells exposed to Mn, 14,15‐EETs, and/or AUDA. (*n* = 3). Data in (F), (G), (H), and (I) are shown as the mean ± SD; data in all other panels are shown as the mean ± SEM. Data in (A), (B), (C), (D), (E), (H), and (I) were analyzed using an unpaired two‐tailed Student's *t*‐test; data in (J), (K), and (L) were analyzed using one‐way ANOVA followed by Tamhane's T2; **p < 0.01, ***p < 0.001.

The MAO‐B mRNA levels were decreased by CYP1B1 overexpression in SH‐SY5Y cells, compared with the control (Figure 5A); additionally, CYP1B1 overexpression markedly up‐regulated the NRF2 mRNA levels (Figure 5B). A previous study has shown the activation of the MAO‐B promoter by KLF11 (TIEG2) in neuronal cells.^29^ A positive correlation between KLF11 and MAO‐B after ethanol exposure was found in both human neuroblastoma SH‐SY5Y cells and rat brains.^30^, ^31^ We found the up‐regulation of KLF11 in the striatum from *Cyp1b1* ^−/−^ mice (Figure 5C), while the transcription factors including SP1, SP3, EGR1, KLF11, HIF1α, JUN, KLF7, FOXC1, PAX6, RAR, RXR, USF1, AP4, and YY1 that are reported or predicted to be involved in the regulation of MAO‐B were unchanged (data not shown). CYP1B1 overexpression reduced KLF11 mRNA levels in SH‐SY5Y cells, compared with the control (Figure 5D); meanwhile, KLF11 overexpression increased MAO‐B mRNA levels (Figure 5E).

EETs from the AA metabolizing pathway have been shown to protect mitochondrial from oxidative stress damage and improve function.^9^ EETs are further metabolized to less active DHETs by soluble epoxide hydrolase, which regulates the intracellular levels of the EETs. The concentration of 14,15‐EET was highest among EET metabolites in the striatum from the wild‐type mice (Figure 5F). CYP1B1 deficiency resulted in decreases in both EETs and DHETs in the striatum (Figure 5G).

The elevation of KLF11 and MAO‐B mRNA levels by Mn exposure was observed in neuronal cells; however, the up‐regulation of KLF11 and MAO‐B by Mn was attenuated by 14,15‐EETs and the soluble epoxide hydrolase inhibitor AUDA (Figure 5H,I). The NRF2 mRNA levels were reduced by Mn exposure in neuronal cells; however, the inhibition of NRF2 by Mn was attenuated by 14,15‐EETs and AUDA (Figure 5J).

### 3.6 Biotin attenuates Mn‐induced dopaminergic damage via up‐regulation of CYP1B1

**FIGURE 6:**
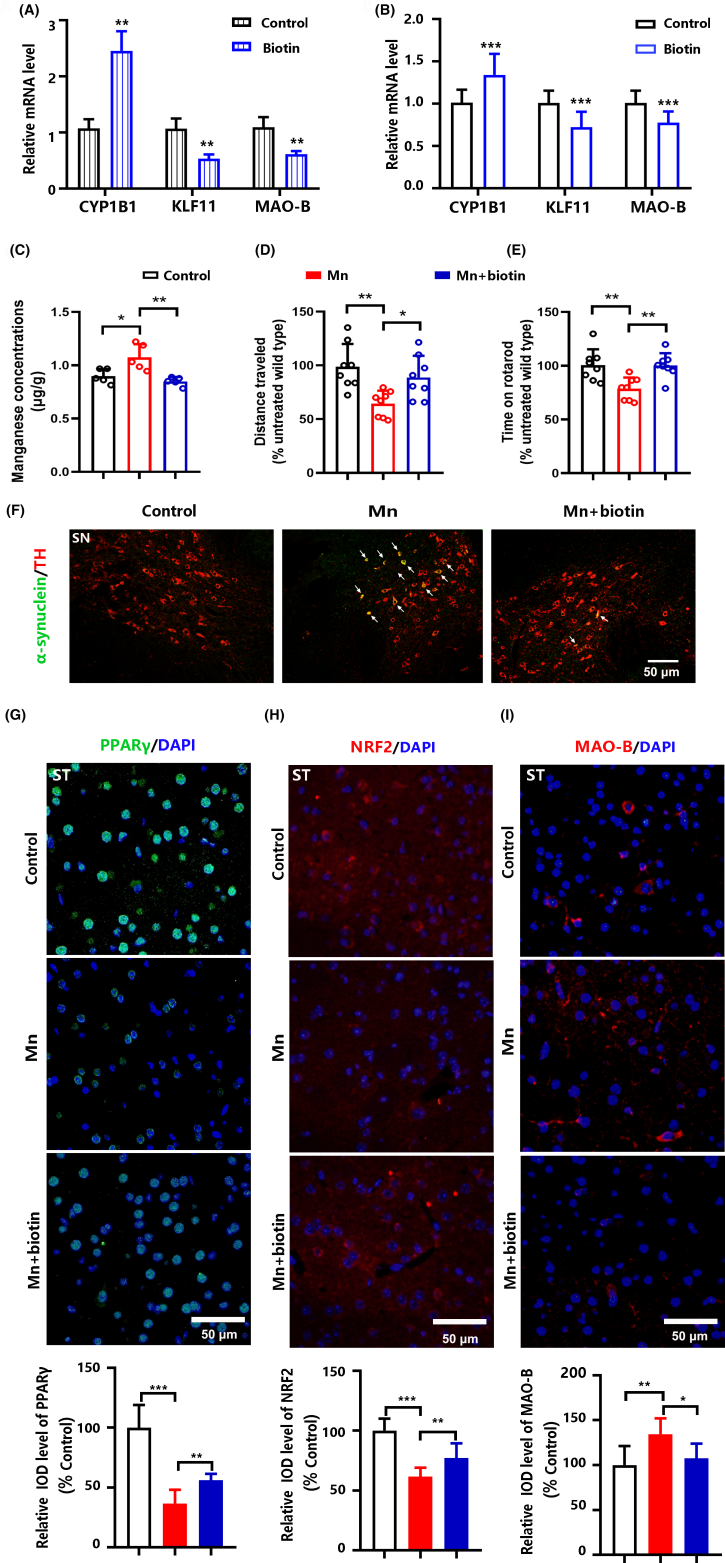
Biotin protects against Mn‐induced damage to dopaminergic neurons in 2‐month‐old mice. (A) Relative mRNA levels of CYP1B1, KLF11, and MAO‐B in SH‐SY5Y cells treated with biotin. (B) Relative mRNA levels of CYP1B1, KLF11, and MAO‐B in the striatum treated with biotin (*n* = 5). (C) Brain Mn concentration in mice intranasally exposed to Mn for 21 days (*n* = 5). (D) The total distance traveled within 5 min in the open‐field test (*n* = 8). (E) Time spent on the rotarod in rotarod test (*n* = 8). (F) Representative images of α‐synuclein (green) and tyrosine hydroxylase‐positive (TH) neurons (red) in the substantia nigra (SN) (*n* = 3). (G‐I) Representative images of PPARγ, NRF2, and MAO‐B proteins in the striatum (ST) (*n* = 3). Data in (A) are shown as the mean ± SEM; data in all other panels are shown as the mean ± SD. An unpaired two‐tailed Student's *t‐*test was used to compare the means between two groups, and one‐way ANOVA followed by the least significant difference (LSD) was used to identify the differences among three groups; *p < 0.05, **p < 0.01, ***p < 0.001.

Biotin is a water‐soluble vitamin that serves as a cofactor for enzymes involved in carboxylation reactions and may regulate protein levels through non‐classical ways such as biotinylation of histones and triggering signal transduction. Biotin markedly up‐regulated CYP1B1 mRNA levels compared with the control, and down‐regulated the mRNA levels of KLF11 and MAO‐B (Figure 6A). Consistent with cellular data, the increase in CYP1B1 mRNA levels and the decreases in KLF11 and MAO‐B levels were also observed in the striatum after biotin treatment (Figure 6B). Mn concentrations in the whole brain tissue from the mice co‐treated with Mn and biotin were lower than those of the mice exposed to Mn alone (Figure 6C); meanwhile, the reduced locomotor activity and motor balance capability by Mn exposure were attenuated in mice co‐treated with biotin (Figure 6D,E). The Mn‐induced accumulation of α‐synuclein was decreased in the striatum from the mice co‐treated with biotin compared with the mice treated with Mn alone (Figure 6F). Mn exposure decreased the protein levels of PPARγ and NRF2 in the striatum; however, the mice co‐treated with Mn and biotin had higher levels of these proteins compared with Mn treatment alone (Figure 6G,H). Additionally, the induction of MAO‐B protein by Mn was decreased in mice co‐treated with biotin (Figure 6I).

## 4 DISCUSSION

The present study provides direct evidence that Mn inhibits brain CYP1B1 and that CYP1B1 deficiency exacerbates Mn‐induced neurotoxicity. CYP1B1 deficiency accelerates Mn accumulation in brain tissue and aggravates the damage to dopaminergic neurons. Brain CYP1B1 deficiency results in the disruption of BBB integrity and oxidative stress in the striatum. Our data firstly demonstrate that biotin attenuates Mn‐induced neurotoxicity in mice, which may be owing to the improvement of BBB hyperpermeability and oxidative stress by the up‐regulation of CYP1B1.

Excessive Mn exposure reduced BBB tight junctions in mice. BBB disruption and reduction in cerebral blood flow were found in rats intraperitoneally injected with Mn nanoparticles (30–40 nm size).^32^ The interplay between brain CYP1B1 and Mn may result in a sustained increase in BBB permeability. BBB disruption has been observed in both patients with Parkinson's disease and MPTP‐induced animal model.^33^, ^34^ Previous studies have shown that a dose‐dependent decrease in β‐catenin‐positive cells in rat striatum by Mn exposure,^35^ and *Cyp1b1* was one of the most prominent genes differentially expressed in β‐catenin‐deficient endothelial cells.^14^ The inhibition of CYP1B1 by Mn might be due to the down‐regulation of β‐catenin signaling. The BBB permeability was increased in mice after subcutaneous injection with CYP1B1 inhibitor, but the permeability of kidney vessels was unchanged.^14^ CYP1B1 deficiency impaired capillary morphogenesis in the retinal endothelial cells.^36^ Our data suggest that CYP1B1 deficiency reduced the length of microvessels and BBB integrity within the brain.

Biotin treatment reduced Mn accumulation in brain tissue during excessive Mn exposure, which may be due to the reduction of BBB permeability via the up‐regulation of CYP1B1. The induction of brain CYP1B1 by biotin is consistent with the previous finding that biotin increased CYP1B1 mRNA levels in human lymphocytes.^37^ Compared with the pretreatment, CYP1B1 ranked fourth among 139 up‐regulated genes in peripheral blood mononuclear cells from healthy adults after biotin supplementation.^38^

CYP1B1‐mediated endogenous metabolites reduced Mn‐induced damage to BBB and oxidative overstress. CYP1B1‐derived HETEs from AA attenuated Mn‐induced down‐regulation of tight junctions via the enhancement of PPARγ transcriptional activity in endothelial cells. PPARγ overexpression in hCMEC/D3 cells markedly reduced HIV‐induced hyperpermeability via up‐regulation of tight junction proteins.^39^

Brain CYP1B1 is responsible for approximately 45% of 14,15‐EET production in the striatum, as indicated by the data from CYP1B1 knockout mice. We found that EETs might be involved in the regulation of dopaminergic homeostasis via the KLF11/MAO‐B axis. The up‐regulation of MAO‐B by CYP1B1 deficiency resulted in increases in the transformation of DOPAC from dopamine in the striatum. 14,15‐EET improved Mn‐induced mitochondrial oxidative stress, possibly due to the anti‐oxidative effect via the up‐regulation of NRF2 or the decreased ROS levels via the inhibition of MAO‐B. EETs have been documented as neuroprotective substrates against ischemic brain damage, neural pain, and inflammation.^40^ Biotin treatment attenuated Mn‐induced up‐regulation of MAO‐B and down‐regulation of NRF2 in mice.

## 5 CONCLUSIONS

Brain CYP1B1 affects blood–brain barrier integrity and dopamine homeostasis, and CYP1B1‐derived AA metabolites attenuate Mn‐induced disruption of tight junctions and oxidative stress. Our data suggest that biotin may attenuate Mn‐induced neurotoxicity by up‐regulation of brain CYP1B1, and that brain CYP1B1 may serve as a novel target for the prevention of neurotoxicity induced by exogenous substances.

## AUTHOR CONTRIBUTIONS

Juan Wu, Yuerang Li, Shuwei Tian, Shufang Na, and Jiang Yue designed the research. Juan Wu, Yuerang Li, Shuwei Tian, Shufang Na, Hongyan Wei, Yafei Yang, Zixia Shen, Jiayue Ding, Shenglan Bao, Siqi Liu, Lingyun Li, and Rongling Feng performed the experiments. Juan Wu, Yuerang Li, Shuwei Tian, Shufang Na, Hongyan Wei, Yafei Wu, Yong Zhu, Chunyan He, and Jiang Yue analyzed the data. Jiang Yue wrote the article. All the authors have read and approved the final manuscript. Juan Wu, Yuerang Li, Shuwei Tian, Shufang Na, and Hongyan Wei contributed equally to this work.

## CONFLICT OF INTEREST STATEMENT

The authors declare no conflicts of interest.

## Supporting information


Table S1.

## Data Availability

The data that support the findings of this study are available from the corresponding author upon reasonable request.
