# Supplementary material for: CYP1B1 affects the integrity of the blood–brain barrier and oxidative stress in the striatum: An investigation of manganese‐induced neurotoxicity
Source: CNS Neurosci Ther. 2024 Mar 1;30(3):e14633. doi: 10.1111/cns.14633 (PMC10907825; doi:10.1111/cns.14633)
Supplement: Supplementary file 1 — Table S1. [file CNS-30-e14633-s001.docx]

# Table S1 Primer sequences used for quantitative real-time RT-PCR

| Gene | Forward /  Reverse | Primer （5’ to 3’） | Tm （℃） |
| --- | --- | --- | --- |
| hCYP1B1 | Forward | GCTGCAGTGGCTGCTCCT | 60 |
|  | Reverse | CCCACGACCTGATCCAATTCT | 60 |
| hGAPDH | Forward  Reverse | CATCACCATCTTCCAGGAGCGAGA  TGCAGGAGGCATTGCTGATGATCT | 60  60 |
| hNRF2 | Forward  Reverse | TCCAGTCAGAAACCAGTGGAT  GAATGTCTGCGCCAAAAGCTG | 60  60 |
| hKLF11 | Forward  Reverse | GCATGACAGCGAAAGGTCTAC  GGGGTCTTATCCGCAACAGG | 60  60 |
| hMAO-B | Forward | GGAGCTAGGATTGGAGACCTAC | 60 |
|  | Reverse | CCCTGAAGGGGTATGATTTGC | 60 |
| hPPARγ | Forward  Reverse | AGTGGGGATGTCTCATAATGCC  AGGTCAGCGGACTCTGGATTC | 60  60 |
| hZO-1 | Forward  Reverse | CAACATACAGTGACGCTTCACA  CACTATTGACGTTTCCCCACTC | 60  60 |
| hOCLN | Forward  Reverse | GACTTCAGGCAGCCTCGTTAC  GCCAGTTGTGTAGTCTGTCTCA | 60  60 |
| hCLDN1 | Forward  Reverse | CCTCCTGGGAGTGATAGCAAT  GGCAACTAAAATAGCCAGACCT | 60  60 |
| mGAPDH | Forward  Reverse | AGGTCGGTGTGAACGGATTTG  TGTAGACCATGTAGTTGAGGTCA | 60  60 |
| mNRF2 | Forward | TCTTGGAGTAAGTCGAGAAGTGT  GTTGAAACTGAGCGAAAAAGGC | 60 |
|  | Reverse |  | 60 |
| mCYP1B1 | Forward | CAGTCTGGCGTTCGGTCAC | 60 |
|  | Reverse | GCTGCGTTGGATCGAGGAA | 60 |
| mPPARγ | Forward  Reverse | TATGGAGTGACATAGAGTGTGCT  CCACTTCAATCCACCCAGAAAG | 60  60 |
| mZO-1 | Forward  Reverse | AGAGGAAACGCCTGTGTGAG  ACGACATACTCAGCACCAGCATCA | 60  60 |
| mOCLN | Forward  Reverse | TTGAAAGTCCACCTCCTTACAGA  CCGGATAAAAAGAGTACGCTG | 60  60 |
| mCLDN1 | Forward  Reverse | GGGGACAACATCGTGACCG  AGGAGTCGAAGACTTTGCACT | 60  60 |
| mMAO-A | Forward | GCCCAGTATCACAGGCCAC | 60 |
|  | Reverse | CGGGCTTCCAGAACCAAGA | 60 |
| mMAO-B | Forward  Reverse | AACAAAAGCGATGTGATCGTGG  GCCCAACATAAGATCCTCCAAGG | 60  60 |
| mCOMT | Forward | CTGGGGCTTGGTGGCTATTG | 60 |
|  | Reverse | CTTTTGCGTCACCCACGTTC | 60 |
| mKLF11 | Forward  Reverse | CATGGACATTTGTGAGTCGATCC  CCTTTGGTAGATCAGGTGCAG | 60  60 |
| mSOD1 | Forward  Reverse | AACCAGTTGTGTTGTCAGGAC  CCACCATGTTTCTTAGAGTGAGG | 60  60 |
| mCAT | Forward  Reverse | TGGCACACTTTGACAGAGAGC  CCTTTGCCTTGGAGTATCTGG | 60  60 |
| mGPX1 | Forward | AATGTCGCGTCTCTCTGAGG  TCCGAACTGATTGCACGGG | 60 |
|  | Reverse |  | 60 |
